# Protection against highly pathogenic SIV by BCG-SIV recombinant priming and attenuated replicating vaccinia-SIV recombinant boosting

**DOI:** 10.1186/1742-4690-9-S2-P39

**Published:** 2012-09-13

**Authors:** X Zhang, H Sakawaki, T Miura, T Igarashi, S Horibata, K Yokomizo, K Matsuo, N Yamamoto, IB Fofana, W Johnson, T Ohashi, H Shida

**Affiliations:** 1 Institute for Genetic Medicine, Hokkaido University, Sapporo, Japan; 2 Institute for Virus Research, Kyoto University , Kyoto, Japan; 3 Japan BCG Laboratory , Tokyo, Japan; 4 National University of Singapore, Japan; 5 NEPRC, Harvard Medical School; Boston College , Boston, USA

## Background

We constructed recombinants of the Japanese-licensed Tokyo 172 strain of BCG and the replication-competent vaccinia virus strain LC16m8Δ (m8Δ) (a genetically stable variant of Japanese licensed smallpox vaccine LC16m8) to express SIV genes. We then evaluated the protective efficacy of these recombinants against challenge with pathogenic, neutralization resistant SIVmac251.

## Methods

Indian rhesus macaques were immunized with rBCG-SIV expressing SIV Gag, Env, or Rev-Tat-Nef (RTN) fusion proteins via subcutaneous injection, followed with two boosts with the Gag-, Env-, RT-, or RTN-expressing m8Δ by skin scarification. Eight weeks after the 2nd boost, macaques were challenged up to 5 times with a low dose of SIVmac251 by the rectal route. Cellular and humoral immune responses were analyzed by standard methods. Plasma SIV RNA and cell-associated SIV proviral DNA in peripheral blood and various tissues were monitored by quantitative PCR.

## Results

Env binding antibodies were elicited at similar levels in all vaccinated macaques after m8Δ boost, but neutralizing Ab against SIVmac239 was not detected. Robust SIV-specific CD4+ and CD8+ effecter memory T cell responses were elicited and maintained at high level until SIV challenge. One vaccinated animal had potent CD8+ T cells that suppressed SIVmac239 replication in vitro. Plasma viraemia was not detected in this animal, even after CD8+ T cell depletion throughout the follow-up period. Protection was confirmed by lack of detectable cell-associated provirus in various organs. A second vaccinated monkey became infected, but viral load remained from one to two logs lower than control monkeys.

## Conclusion

Vaccine-induced SIV specific T cell responses appear to be effective against SIV challenge. Importantly, our results suggest that a vaccine regimen based on an rBCG prime and vaccinia m8Δ boost (both liscensed vaccine platforms with a long track record of safety in humans) should be explored as a safe and valuable means for efficacious HIV/AIDS vaccine.

